# Impact of intragastric administration of donkey milk on mouse immunity utilizing gut microbiomics and plasma metabolomics

**DOI:** 10.3389/fvets.2025.1486406

**Published:** 2025-03-12

**Authors:** Jianwen Wang, Wanlu Ren, Zhiwen Sun, Shibo Liu, Zixiang Han, Yongfa Wang, Yaqi Zeng, Jun Meng, Xinkui Yao

**Affiliations:** ^1^College of Animal Science, Xinjiang Agricultural University, Ürümqi, China; ^2^Xinjiang Key Laboratory of Equine Breeding and Exercise Physiology, Ürümqi, China

**Keywords:** gut microbiome, metagenomics, metabolomics, immunity, donkey milk

## Abstract

**Introduction:**

Donkey milk demonstrates closer compositional resemblance to human milk compared to bovine milk, positioning it as an optimal nutritional substitute for infants with cow's milk allergy. Furthermore, its rich profile of bioactive compounds suggests potential immunomodulatory properties. This study systematically investigated the effects of donkey milk supplementation on murine immune function and gut microbiome dynamics, thereby providing mechanistic insights to support its clinical development in functional food applications.

**Methods:**

Following daily intragastric administration of 10 mL/kg of body weight of donkey milk (DM) or distilled water (DW) to the mice for 28 consecutive days, liver tissues were harvested for immunological profiling, with concurrent collection of blood samples for plasma metabolomic analysis and fecal specimens for gut microbiome characterization. Subsequently, the modulatory effects of donkey milk supplementation on immune parameters, intestinal microbiota composition, and plasma metabolic profiles were systematically evaluated.

**Results:**

Immunity analysis revealed that intragastric administration of DM raised the levels of IL-6 and TNF-α cytokines in mouse liver. In addition, DM modulated the composition of both the murine gut microbiome and plasma metabolites. One-hundred and forty-five differentially-produced metabolites were identified, most prominently nicotinamide, L-valine, and β-estradiol, that are primarily associated with valine, leucine, and isoleucine biosynthesis and degradation, nicotinate and nicotinamide metabolism, and unsaturated fatty acid biosynthesis. Alterations at phylum, genus, and species levels were evident in the fecal microbiota of mice after intragastric administration of DM. In particular, an increased abundance of the *Lactobacillus* bacterium was observed. Correlation analysis of differential metabolites and microbiomes indicated a correspondence between *Falsiroseomonas* and *Salipiger* species and the antioxidant coenzyme Q that has the potential to activate the immune system.

**Conclusion:**

The data collectively suggest that DM may adjust the murine gut microbiome and plasma metabolites thereby potentially improving immunity in mice.

## 1 Introduction

Milk and dairy products play critical roles in human diet and nutrition. Breast milk is the main nutritional source for newborns during early life stages due to the rich content of milk nutrients. As well as providing essential nutrients for growth and development, milk also improves body health (1). Research on milk and dairy products has focused principally on cow milk. However, milks from diverse sources exhibit compositional differences which may affect the biological activities of distinct types of milk (2). Interest in and demand for non-cow milk products are growing in part due to the increase in dairy consumption and the development of enhanced processing technologies (3). Donkey milk (DM) is one potential alternative to cow milk. Elucidation of the bioactive functions and regulatory mechanisms in DM production will facilitate the development of targeted active DM products that respond to the changing needs of different populations for dairy products.

The composition of DM is more similar to human milk than cow milk which renders DM an ideal substitute for infants who are allergic to bovine milk (4). DM has a similar protein content (1.63 g/100 mL) as human milk (1.42 g/100 mL) and is approximately half the content of cow milk (3.25 g/100 mL). In addition, cow milk has a relatively high casein content whereas human milk and DM have comparatively high levels of whey proteins which may explain why DM causes fewer allergies than cow milk (5). Furthermore, β-lactoglobulin in DM may be digested more easily than β-lactoglobulin in cow milk (6). DM has a lower butterfat content than human and cow milks (7). However, DM has a high content of essential fatty acids, including linoleic acid and linolenic acid, which are 25% more than in cow milk (8). Moreover, DM contains abundant water-soluble vitamins, including vitamin B and vitamin C, and fat-soluble vitamins, particularly vitamin D (9), which further emphasizes the high nutritional value of DM.

In addition, DM may exhibit antibacterial properties against foodborne pathogens (10, 11). Numerous bioactive peptides with immune-like properties, including lysozymes and lactoferrin, have been isolated from DM (12) which suggests that DM may play improve immune capacities. We hypothesized that the immunomodulatory effect of donkey milk may arise from its capacity to modulate the human gut microbiome, thereby influencing metabolic processes. We tested our hypothesis by administering donkey milk to mice with subsequent evaluation of immune capabilities. In addition, the relationship between the gut microbiome and serum metabolites of mice was characterized with the aim of identifying biomarkers in DM that improve the immune system which will further guide the production and application of DM as a beneficial food product.

## 2 Materials and methods

### 2.1 Ethical statement

This study was approved by the Animal Welfare and Ethics Committee of Xinjiang Agricultural University (approval number: 2023009).

### 2.2 Experimental animals and sample collection

DM samples were collected from animals in Tacheng, Xinjiang, China, and were divided and placed into sterile polyethylene tubes which were frozen immediately at −20°C. DM originated from four Xinjiang donkeys (*Equus africanus asinus*). The nutritional composition of the DM is provided in Supplementary Table S1. Samples were transferred to the laboratory in an ice bath and then were stored at −20°C for subsequent use. All DM samples came from a single-time collection and were thawed with water before use.

Twenty-eight 4-week-old Institute of Cancer Research (ICR) mice purchased from Xinjiang Medical University (Xinjiang, China) were kept at 22 ± 2°C with a 12 h light/dark cycle. Mice had free access to food and water throughout the experimental period. Mice were divided randomly into two groups after 1 week of acclimation. The animals were housed in groups of three or four per cage (290 × 178 × 160 mm), with males and females maintained separately. The selected mice were fed a standard rodent diet sourced from Sibeifu Biotechnology Co., Ltd. (Beijing, China). The nutritional composition of the feed is provided in Supplementary Table S2. Each group contained seven male mice and seven female mice which were administered intragastric injections every morning of 10 mL/kg of body weight of DM or distilled water (DW) for four consecutive weeks. Mice were weighed on the 1st day of each week to adjust the amount of feed DM and DW. Body weight data for mice is presented in Supplementary Table S3. Animals were fasted for 12 h with no access to water for 6 h after the final feeding on day 28 (13), and then were sacrificed on day 29. During the procedure, the experimenter grasped the mouse tail base with the right hand, lifted it, and placed it on the cage lid or another rough surface. The experimenter then pressed the head and neck with the left thumb and index finger while pulling the tail base backward and upward with the right hand. This procedure resulted in cervical dislocation, severing spinal cord and brain stem, and causing instantaneous death of the animal. The animals were anesthetized with pentobarbital before sacrifice. Livers were subsequently harvested to detect immune indicators. Four male mice and four female mice (two per cage) from each group were selected randomly for collection of blood and fecal samples. Immune biomarker analysis was conducted immediately on the harvested livers. Blood samples were collected in vacuum tubes and allowed to stand at room temperature for 1 h prior to centrifugation at 1,000 g for 10 min to obtain sera which were subsequently stored at −80°C for metabolite detection. Fecal samples were collected using sterile tubes and were frozen at −80°C for microbial analysis.

### 2.3 Immunological analysis

Livers, spleens, and thymuses were harvested, rinsed with saline, and dried with filter paper. The organs were weighed with the spleen and thymus indices calculated as: organ index = organ weight/body weight × 100%. The TNF-α (MM-0132M), IFN-γ (MM-0182M), and IL-6 (MM-0163M) levels in liver samples were measured by ELISA with assay kits purchased from Jiangsu Meimian Industrial Co., Ltd. (Yancheng, China). Assays were conducted according to the manufacturer's instructions.

### 2.4 Serum metabolomics analysis

One hundred microliters of serum sample was thawed at room temperature and transferred to a polyethylene tube. Four hundred microliters of an 80% methanol aqueous solution was added followed by vortexing. The sample was placed on ice for 5 min, after which it was centrifuged at 15,000 g and 4°C for 20 min. The centrifugation step was repeated and the supernatant was collected for LC-MS analysis.

The LC-ESI-MS/MS system from Novartis (Beijing, China) in conjunction with the Hypesil Gold column (100 × 2.1 mm, 1.9 μm, ThermoFisher, USA) was used to conduct untargeted metabolite detection in serum according to previous methodology (14). Analytes were eluted with a velocity gradient of 0.20 mL/min under a column temperature of 40°C using 0.1% formic acid aqueous solution (A) and methanol (B) or 5 mM ammonium formate aqueous solution (C) and methanol (D). After equilibration, 2 μL of the solution were injected into each sample, with the following linear gradients of solvent B (v/v): 0–3 min, 2% B/D; 3–10 min, 85% B/D; 10–10.1 min, 0% B/D; and, 10.1–11 min, 2% B/D. Electrospray ionization mass spectrometry (ESI-MSn) was performed using a Q Exactive™ HF/Q Exactive™ HF-X mass spectrometer (Thermo Fisher) with the following parameters: spray voltage = 3.5 kV; sheath gas flow velocity = 35 psi; auxiliary gas flow velocity = 10 L/min; ion transfer tube temperature = 320°C; auxiliary gas heater temperature = 350°C; and, scanning range = 100–1,500 m/z.

### 2.5 Metabolomics data analysis

Simple screening using Compound Discover (CD) 3.3 software with parameters of retention time and mass-to-charge ratio was performed on raw data of plasma metabolomics with peak areas quantified. In addition, identified metabolites were annotated using KEGG, HMDB, and LIPIDMaps databases. Partial least squares discriminant analysis (PLS-DA) was performed on data using the metaX software, thereby obtaining the Variable Importance in the Projection (VIP) value of each metabolite. The statistical significance (*P* value) of each metabolite was calculated on the basis of the *t*-test with fold change (FC) values determined between the two groups. The fold change (FC) is calculated using the formula:


(1) $$\begin{array}{l} FC=FC=x_{\text{DW}}/x_{\text{DM}}, \end{array}$$

where x_DW_ and x_DM_ represent the relative quantification values for plasma metabolites in the DW and DM groups, respectively.

Screening of differential metabolites and clustering analysis were performed based on the criteria of VIP>1, FC>1.2 (or FC < 0.833), and *P* < 0.05. Metabolic pathway analysis using the KEGG database was conducted on differential metabolites that were identified.

### 2.6 Metagenomic analysis of gut contents

Genomic DNA was extracted from the gut contents of mice. DNA concentrations were measured using a Qubit^TM^ dsDNA Assay Kit (Invitrogen, Beijing, China, Q32854) and a Qubit^TM^ 2.0 Fluorometer (Invitrogen), and DNA purity and integrity were assessed by 1% agarose gel electrophoresis. DNA libraries were established using the NEBNext^TM^ Ultra DNA Library Prep Kit for Illumina (New England Biolabs). Libraries were diluted to 2 ng/μL and insert sizes were measured using an Agilent 2100 bioanalyzer. Sequencing on the Illumina PE150 platform was performed after the effective concentrations of the libraries were quantified by Quantitative Real-Time PCR (Q-PCR).

### 2.7 Metagenomic data analysis

Data analysis was performed as outlined elsewhere (15). Briefly, raw sequencing data were processed using Readfq (https://github.com/cjfields/readfq) to remove adaptor contaminants and reads with low-quality bases exceeding 40 nt (threshold < 38), reads with N bases >10 nt, and reads with adapter overlap >15 nt. Bowtie2 (http://bowtie-bio.sourceforge.net/bowtie2/index.shtml) was used to filter reads derived from the mouse host. Clean data subsequently were assembled using MEGAHIT (1.2.8) and assembled scaffolds were broken at the N connection points with Scaftigs not containing N obtained. An Open Reading Frame (ORF) prediction was conducted on Scaftigs samples (≥500 nt) using MetaGeneMark (http://topaz.gatech.edu/GeneMark/). Information with a length shorter than 100 nt among prediction results was filtered out, and redundant results were removed using CD-HIT (http://www.bioinformatics.org/cd-hit/). Bowtie2 was then used to compare the clean data of each sample with the initial gene catalog and the number of reads of matched genes in each sample was calculated. With the removal of genes with count numbers of no more than two in each sample, a final gene catalog (unigenes) was obtained for subsequent analysis.

Unigenes were compared and annotated against the sequences of the NCBI NR (https://www.ncbi.nlm.nih.gov/) and KEGG databases (http://www.kegg.jp/kegg/) using DIAMOND (https://github.com/bbuchfink/diamond/). Lowest Common Ancestor (LCA) algorithm in MEGAN 6 was applied to perform species classification and annotation. The abundance information of each sample was obtained from the LCA annotation results and gene abundance table. Anosim analysis (R vegan package, 2.7-0) was performed to measure differences between groups. In addition, differences in the relative abundance of species and KEGG pathways were analyzed with linear discriminant analysis effect size (LefSe) (http://galaxy.biobakery.org/) with different microbiomes and functions between the DW and DM groups identified.

### 2.8 Integrated metagenomic and metabolomics analysis

The Spearman rank correlation method was applied for correlation analysis between the microbiomics and metabolomics data. An *R*-value >0.5 or <-0.5 indicated a strong correlation and *P* < 0.05 indicated a significance level. Correlation heat maps were generated using the “Complex Heat Map” package in R language.

### 2.9 Statistical analysis

An independent sample *t*-test was performed on immunological indicators using the SPSS21 software with *P* < 0.05 indicating a significance level. Graphs were plotted using GraphPad Prism 8.

## 3 Results

### 3.1 Influence of intragastric administration of donkey milk on mouse immune biomarkers

**Figure 1:**
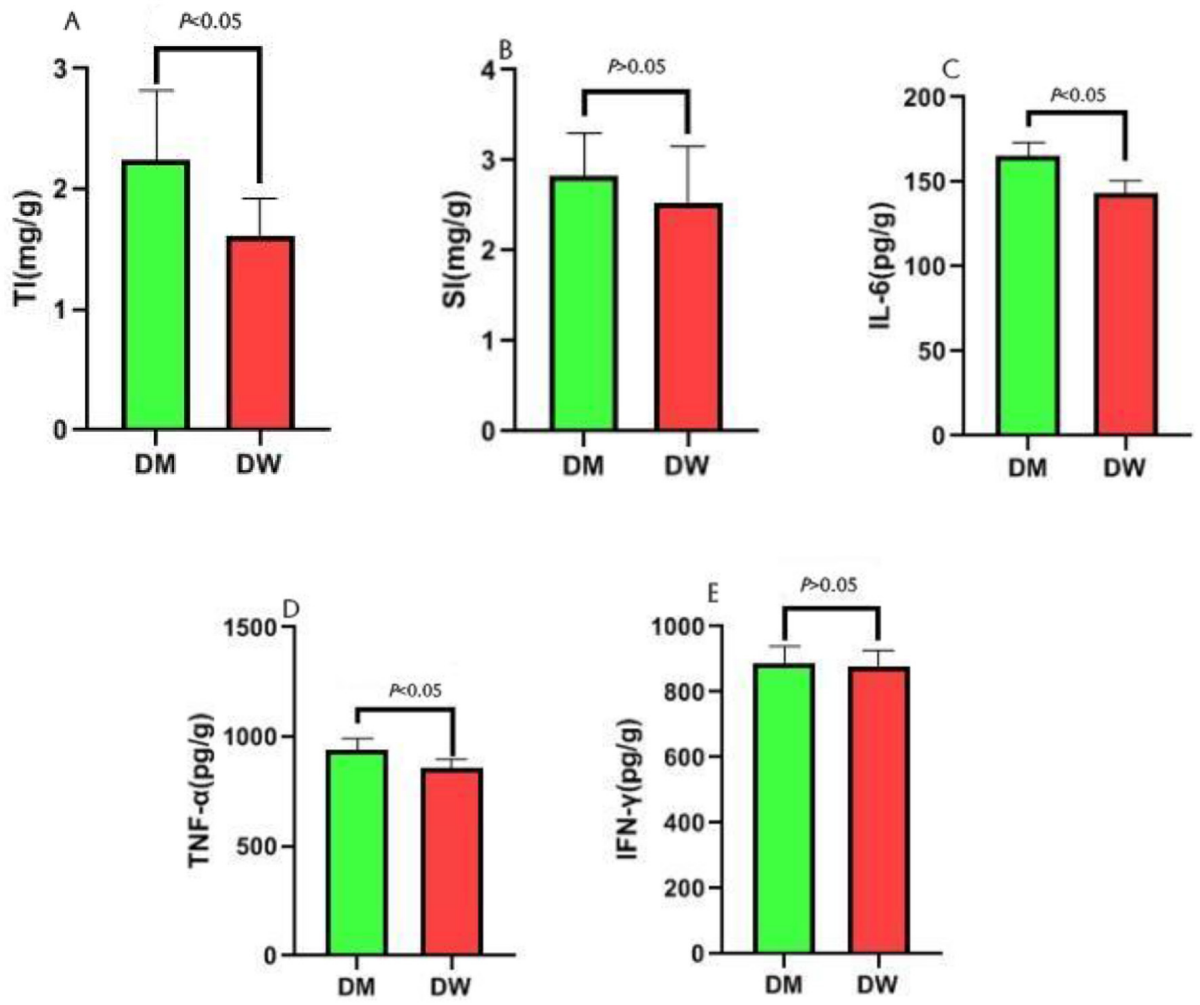
Effect analysis of intragastric administration of DM on mouse immunity. TI is the abbreviation for Thymus index, and SI is the abbreviation for Spleen index. **(A)** Comparison of thymus index between DW group and DM group; **(B)** Comparison of spleen index between DW group and DM group; **(C)** Comparison of IL-6 between DW group and DM group; **(D)** Comparison of TNF-α between DW group and DM group; **(E)** Comparison of IFN-γ between DW group and DM group.

Thymus and spleen indices indicate the sizes of the organs and allow for assessment of potential changes in the immune response following administration of external agents. Compared with mice in the DW group, mice in the DM group exhibit a significantly increased thymus index (*P* < 0.01) (Figure 1A) and an increased spleen index without significant difference (*P* < 0.05) (Figure 1B). ELISA test results show that mice in the DM group present significantly increased levels of IL-6 (Figure 1C) and TNF-α (Figure 1D) (*P* < 0.01) and an increased level of IFN-γ (Figure 1E) with no significant difference (*P* < 0.05). Thus, feeding with DM induces morphological and physiological changes in the murine immune response.

### 3.2 Metabolomics analysis of mouse serum

**Figure 2:**
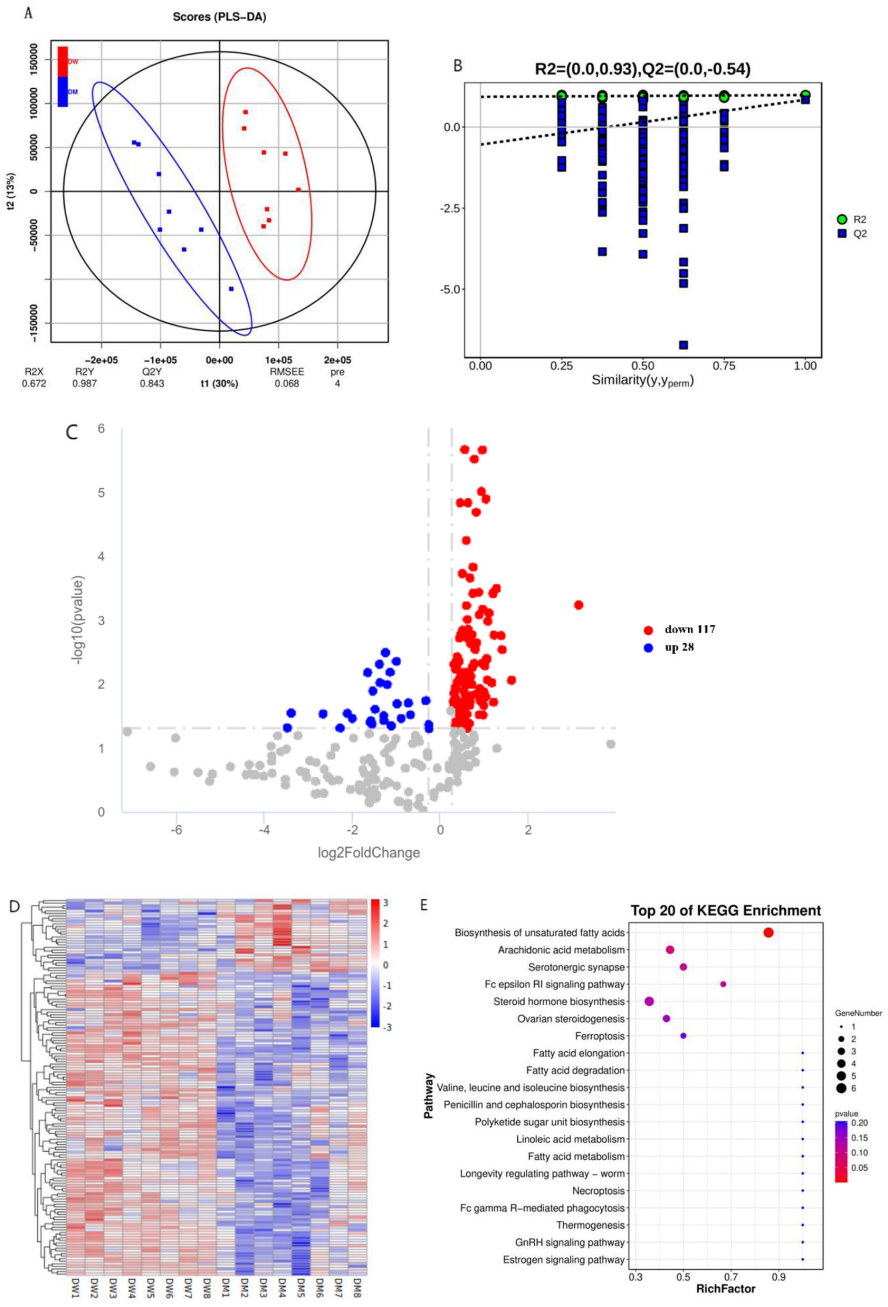
Metabolomics analysis of mice after intragastric administration of DM. **(A)** PLS-DA clustering analysis of mice in the DW and DM groups. **(B)** PLS-DA permutation test. **(C)** Volcano plot of differential metabolites of mice in the DW and DM groups. **(D)** Clustering heat map of differential metabolites of mice in the DW and DM groups. **(E)** KEGG enrichment bubble plot of differential metabolites of mice in the DW and DM groups. *R*^2^ represents the proportion of data variance or dispersion that the current model can explain, while *Q*^2^ reflects the proportion of data variance that the current model can predict.

Partial least squares discriminant analysis (PLS-DA) showed that the serum metabolomics data exhibited significant clustering (Figure 2A). The intercept of the Q2 regression line on the vertical axis is below zero which indicated no presence of overfitting and a reliable PLS-DA model (Figure 2B). A total of 145 differential metabolites were identified under the criteria VIP>1.0, FC>1.2 (or FC < 0.833), and *P* < 0.05. Mice in the DM group exhibited significantly increased levels of 28 differential metabolites and significantly decreased levels of 117 differential metabolites compared with mice in the DW group. These data indicate that intragastric administration of DM alters the serum metabolomics composition of mice (Supplementary Tables S4, S5 and Figures 2C, D). The important differential metabolites mainly include nicotinamide, L-valine, nicotinamide, and β-estradiol.

KEGG enrichment analysis was conducted to determine the metabolic pathways of mice through which the intragastric administration of DM improves immunity. The altered metabolites were associated mainly with the unsaturated fatty acid biosynthesis pathway (Figure 2E and Supplementary Table S5). Thus, the metabolic pathways in mice that are altered following intragastric administration of DM principally involve the metabolism of protein and fat.

### 3.3 Differences in gut microbiomes of mice administered donkey milk

**Figure 3:**
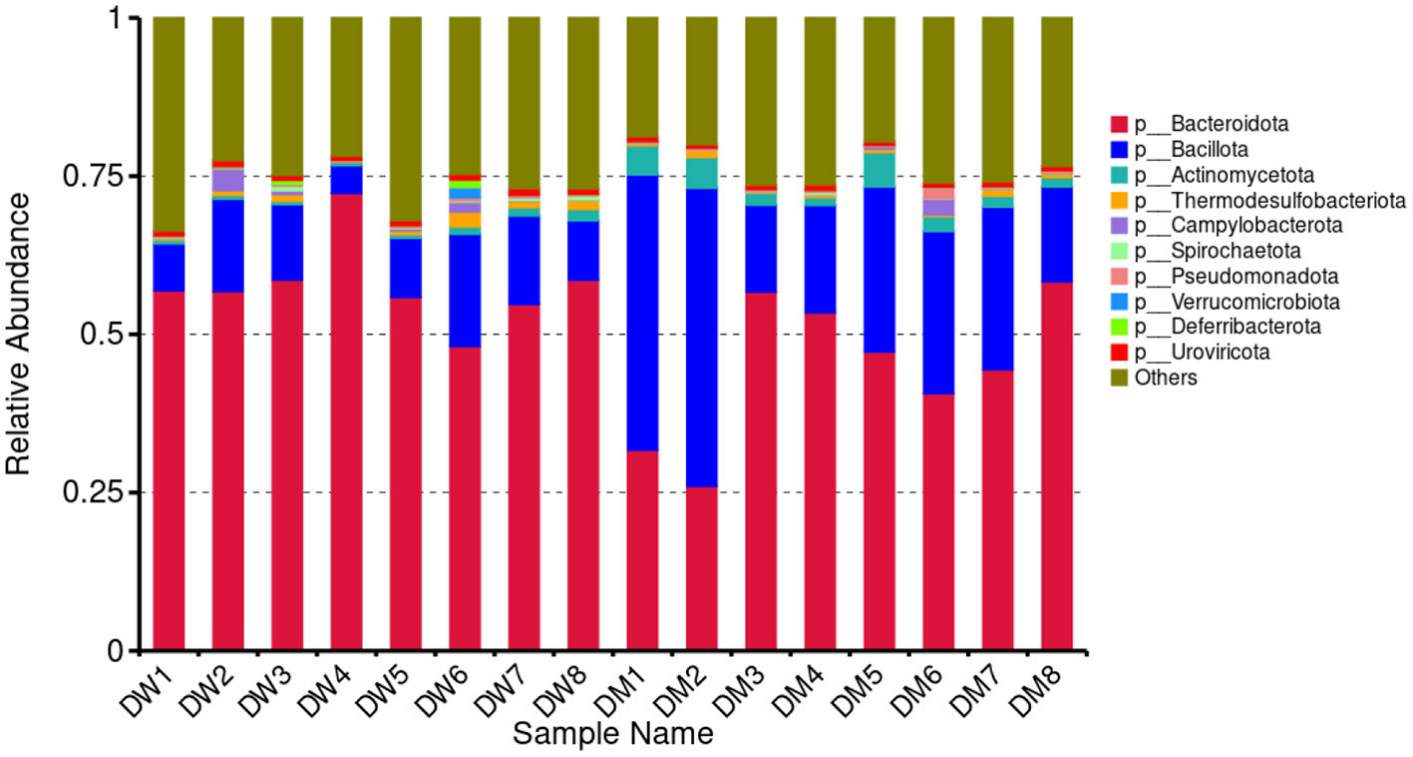
Relative abundance of mouse gut microbiomes at the phylum (p) level.

Metagenomic sequencing and analysis of fecal samples of eight DW and eight DM mice showed the presence of 166 phyla, 142 classes, 256 orders, 559 families, 2,160 genera, and 9,471 microbial species. Based on the species annotation results, the 10 most abundant phyla in each sample were selected to plot a histogram of relative abundance (Figure 3). Gut microbiomes of mice in the DW group at the phylum level primarily consisted of *Bacteroidota* (0.4809–0.7228) and *Bacillota* (0.0435–0.1773), whereas fecal microbiomes of mice in the DM group primarily were members of the *Bacteroidota* (0.2602–0.5831), *Bacillota* (0.1370–0.4709), and *Actinomycetota* (0.0123–0.0544) phyla.

**Figure 4:**
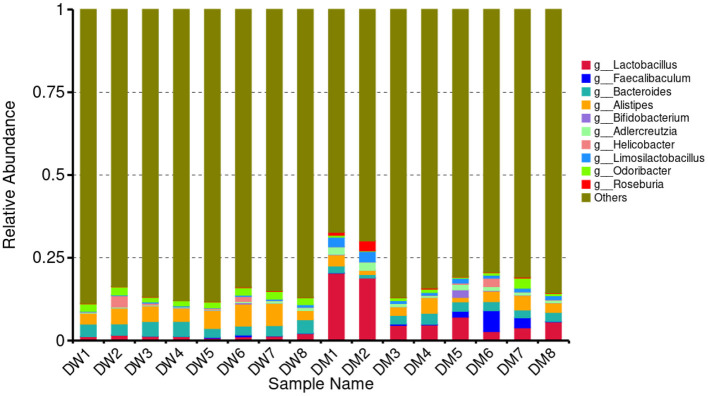
Relative abundance of gut microbiomes at the genus (g) level in DM and DW mice.

LCA was performed to assess significant differences at the genus level between mice in the DW and DM groups (Figure 4). The 10 bacterial genera with the highest relative abundance among mice in the groups were *Lactobacillus, Faecalibaculum, Bacteroides, Alistipes, Bifidobacterium, Adlercreutzia, Helicobacter, Limosilactobacillus, Odoribacter*, and *Roseburia*. Mice in the DM group showed significantly reduced relative abundance of *Bacteroides* and *Odoribacter* (both *P* < 0.01) and *Alistipes* (*P* < 0.05), and significantly increased relative abundance of *Adlercreutzia* and *Limosilactobacillus* (both *P* < 0.01) and *Lactobacillus* (*P* < 0.05) compared with mice in the DW group. No significant differences were observed in the relative prevalence of other bacterial genera.

### 3.4 LEfSe analysis of differential species of the microbiota of mice administered donkey milk

**Figure 5:**
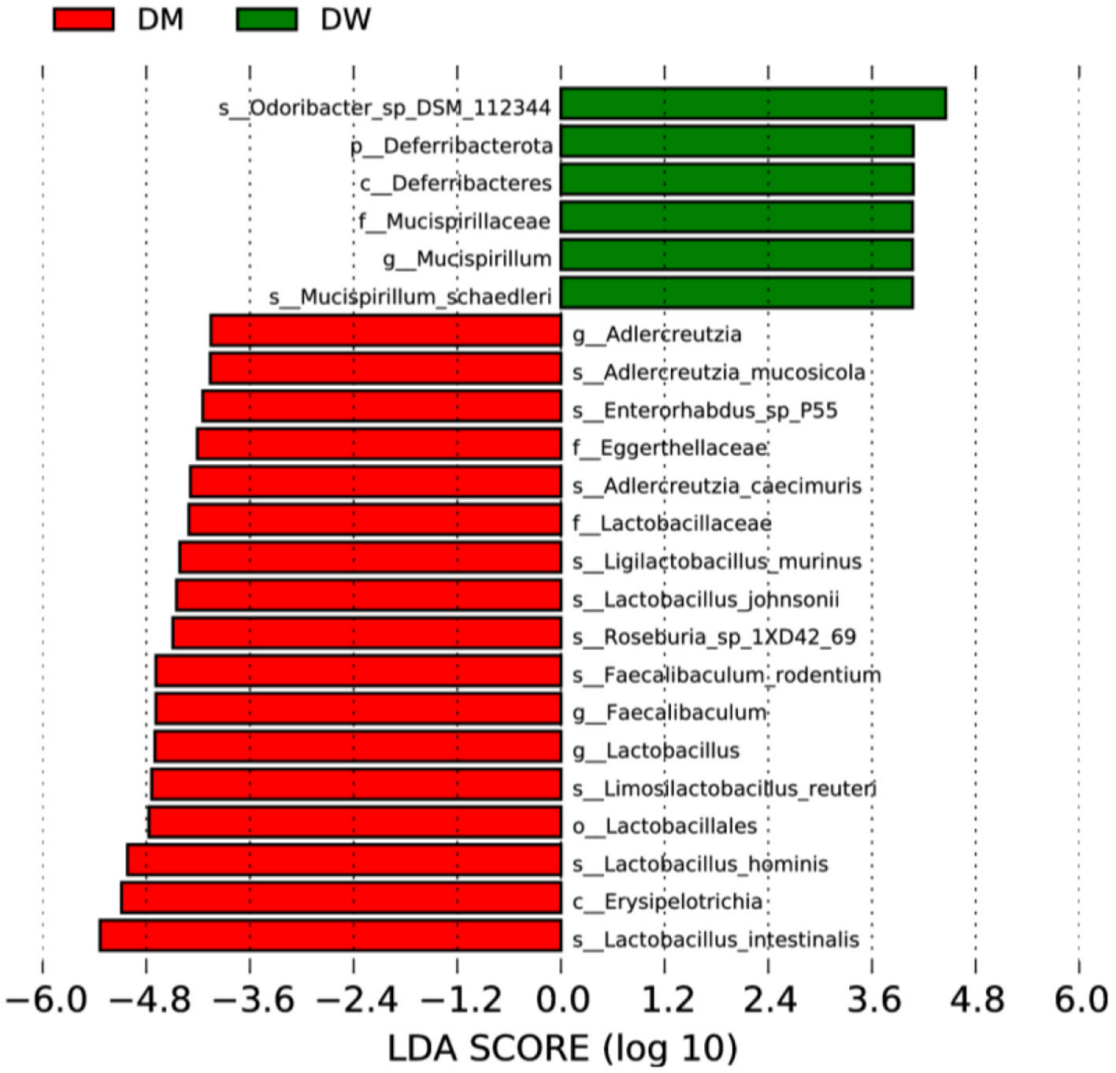
LEfSe analysis of fecal microbiota of mice in the DW (green) and DM (red) groups. p, phylum; s, species; c, class; o, order; f, family; g, genus.

Twenty-four bacterial species were identified by LEfSe analysis that were significantly different in the fecal microbiota of mice in the DW and DM groups (Figure 5). Eighteen species in the fecal microbiota of mice in the DM group were present at significantly increased levels, including *Lactobacillus intestinalis, Erysipelotrichia*, and *L. hominis*, whereas six species in the fecal microbiota of mice in the DW group exhibit significantly increased levels, including *Mucispirillaceae* and *Mucispirillum*. Thus, intragastric administration of DM alters the fecal microbiota of mice at phylum, genus, and species levels.

### 3.5 Functional annotation of mouse gut microbiomes

**Figure 6:**
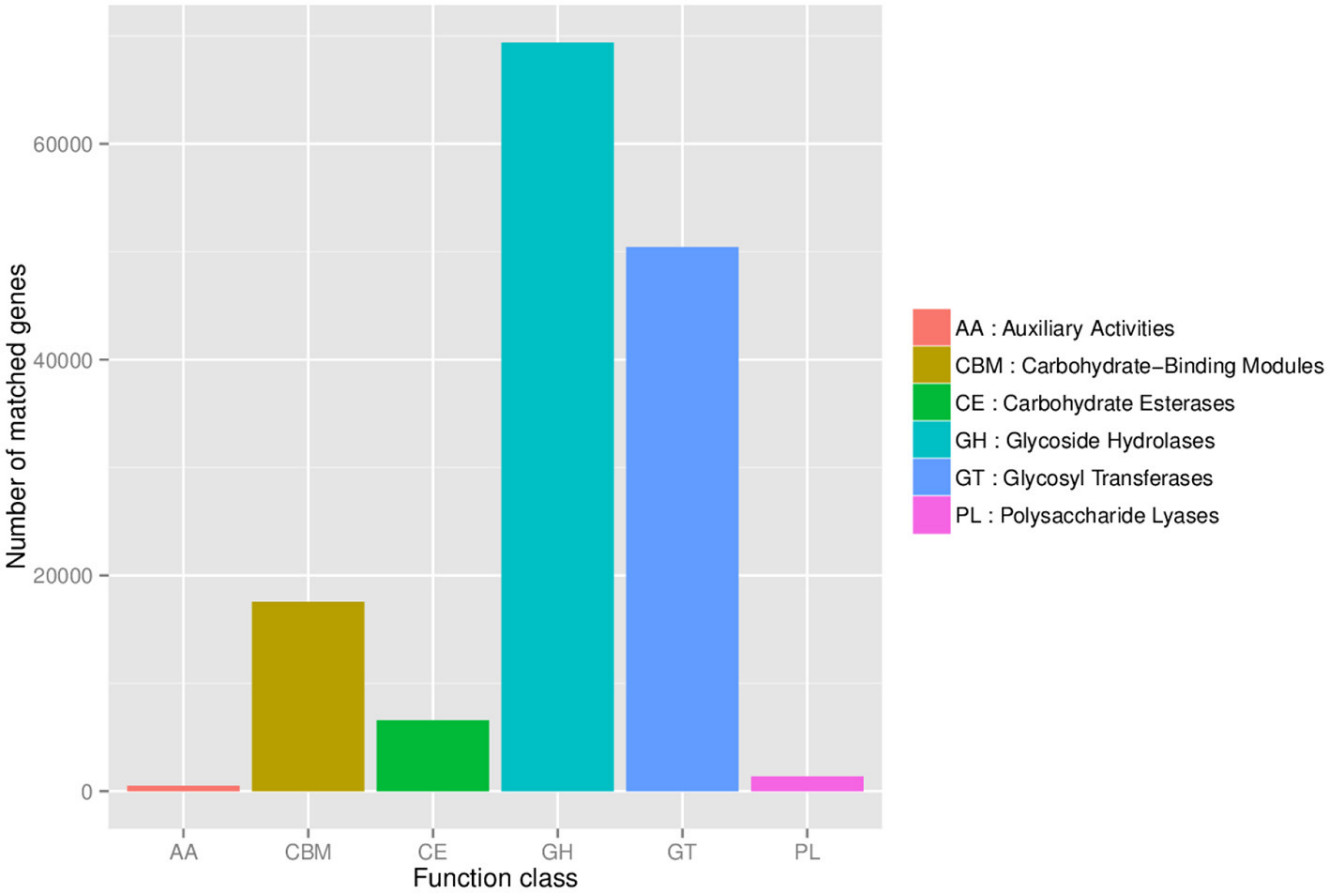
Statistical analysis of carbohydrate-active enzymes in the mouse fecal microbiome.

Statistical analysis of the number of annotated carbohydrate-active genes in the fecal microbiomes of DM and DW mice was plotted with the CAZY database used as a reference (Figure 6). Glycoside hydrolases presented the largest number of genes in the microbiomes followed by glycosyl transferases, carbohydrate-binding modules, and carbohydrate esterases, with polysaccharide lyases and auxiliary activities contributing the fewest genes.

**Figure 7:**
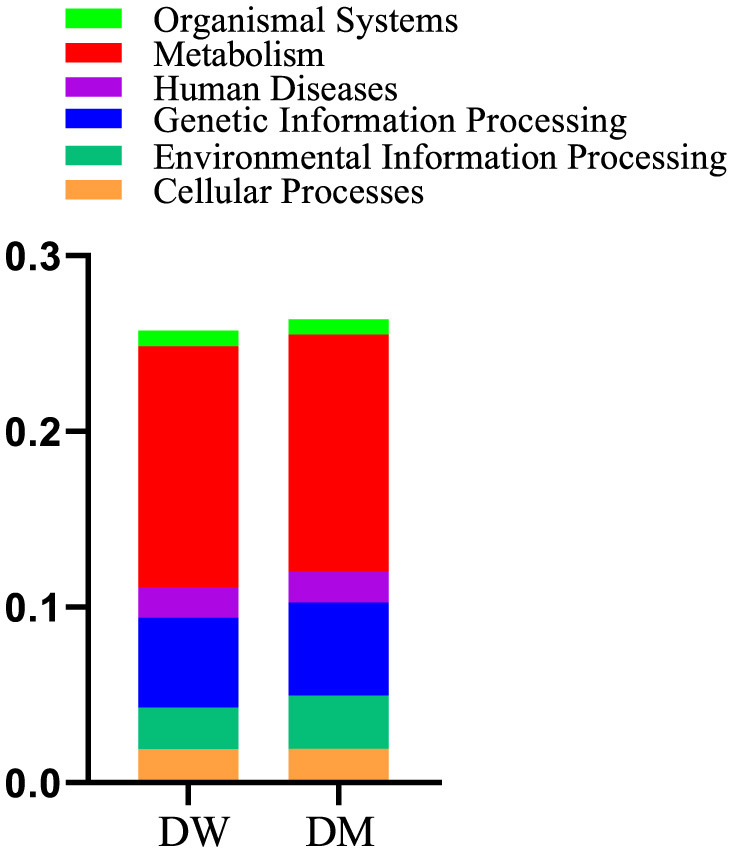
Relative abundance of gene functional annotation at level 1. Level 1 is the first level of the KEGG metabolic pathways which encompasses six major metabolic pathways.

**Figure 8:**
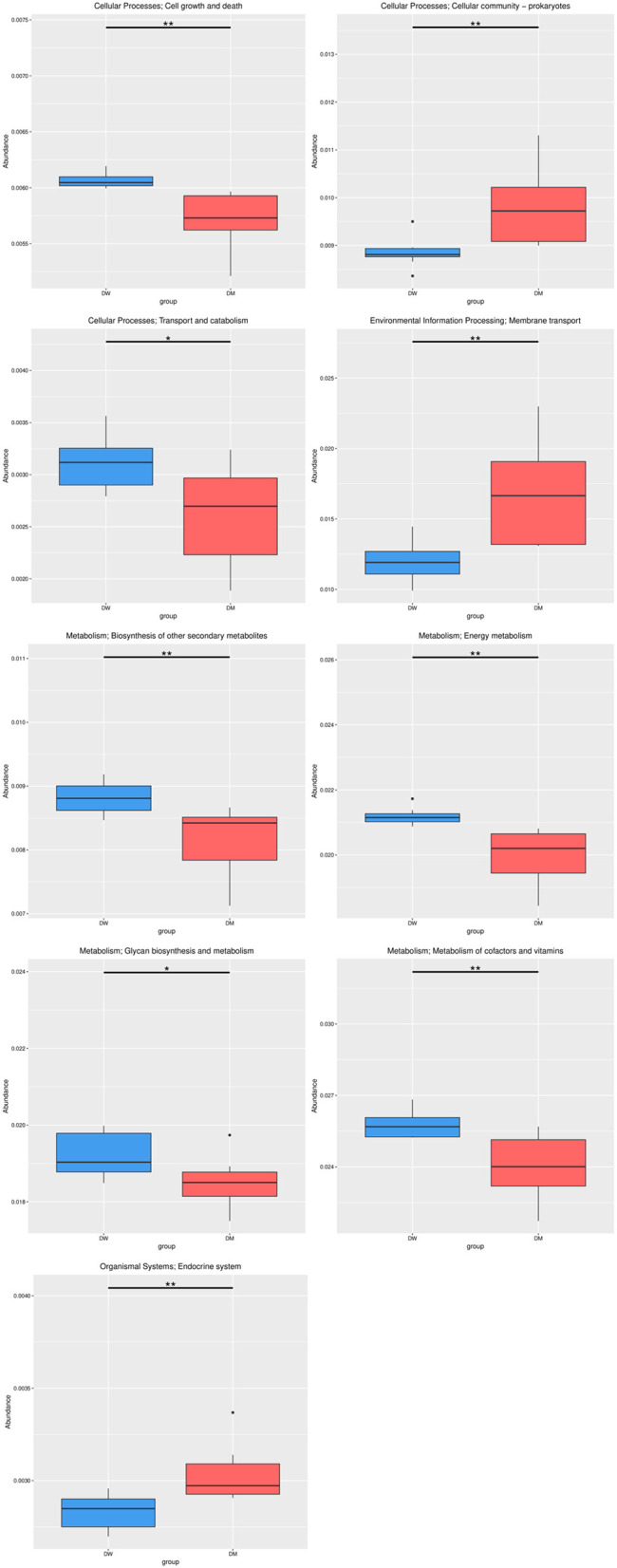
Relative abundance of gene functional annotation at level 2. The most prominent classes of genes in DM and DW mice are shown in red and blue, respectively. Level 2 is the second level of 57 seed pathways in KEGG metabolism. ^*^Indicates *q*-value < 0.05 and ^**^Indicates *q*-value < 0.01.

Metabolism presented the highest number of annotation genes at level 1 according to the annotation results of the KEGG database, with the smallest number of annotation genes presented by organismal systems (Figure 7). Level 2 genes with significantly different relative abundance in DM and DW mice were concentrated primarily in metabolic pathways, including the metabolism of cofactors and vitamins, energy metabolism, glycan biosynthesis, and metabolism and biosynthesis of other secondary metabolites, all of which presented in reduced abundance in the DM group (Figure 8). In addition, genes for cellular processes, most notably cellular community in prokaryotes, showed increased prevalence in the DM group, whereas genes involved in cell growth and death, and transport and catabolism displayed decreased abundance in this group. Furthermore, genes for membrane transport in environmental information processing, human diseases, and the endocrine system within organismal systems all presented with increased abundance in the DM group compared to DW mice.

### 3.6 Correlation analysis between serum metabolites and gut microbiomes after intragastric administration of donkey milk

Pearson correlation analysis was conducted to elucidate the relationship between gut microbiomes and serum metabolites of mice in the DM and DW groups. This analysis also was used to identify the coordinated or opposite changes in gut microbiome structures and serum metabolites of mice after intragastric administration of DM.

**Figure 9:**
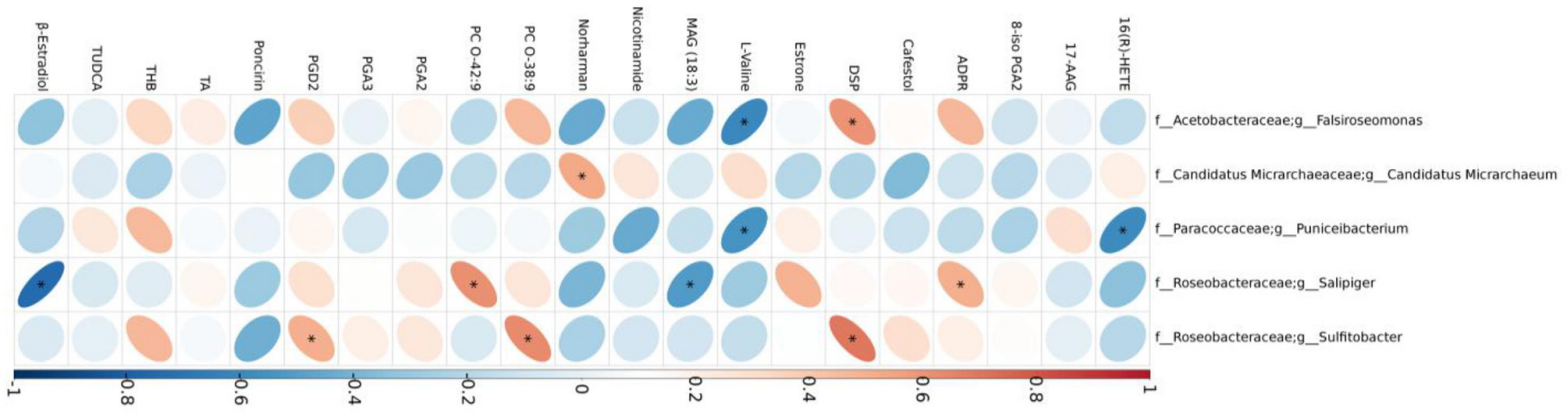
Correlations between gut microbiomes and serum metabolites. TUDCA, taurochenodeoxycholic acid; ADPR, adenosine diphosphate ribose; PGA2, prostaglandin A2; THB, tetrahydrocorticosterone; PGD2, prostaglandin D2; 8-iso PGA2, 8-iso prostaglandin A2; PGA3, prostaglandin A3; DSP, 1-[(3,5-dimethylisoxazol-4-yl)sulfonyl]piperidine; TA, testosterone acetate.

Correlations between five differential bacterial genera and important differential metabolites of mice in the DW and DM groups revealed that the *Falsiroseomonas* genus was significantly and negatively correlated with L-Valine, and positively correlated with 1-[(3,5-dimethylisoxazol-4-yl)sulfonyl]piperidine. *Candidatus Micrarchaeum* was significantly and positively correlated with the beta-carboline alkaloid norharman, whereas the Puniceibacterium genus was significantly and negatively correlated with L-valine and 16(R)-HETE which is a CYP450 metabolite of arachidonic acid. In addition, *Salipiger* was significantly and negatively correlated with monoacylglycerol MAG (18:3) and β-estradiol, and positively correlated with phosphatidylcholine O-42:9 and adenosine diphosphate ribose. Sulfitobacter was significantly and positively correlated with prostaglandin D2, phosphatidylcholine O-38:9 and 1-[(3,5-dimethylisoxazol-4-yl)sulfonyl]piperidine (Figure 9).

## 4 Discussion

DM exerts detectable effects on immunoregulation and increases the levels of cytokines involved in the immune response, including IL-1, IL-6, and TNF-α (16), and also induces the release of the IL-10 anti-inflammatory cytokine (17) in animal model studies. In addition, whey proteins in DM stimulate mouse spleen cells to produce specific immunoregulatory cytokines, including IFN-γ (18). We showed here that mice exhibit increased IL-6 and TNF-α levels after intragastric administration of DM which is consistent with results of previous studies (18). These observations suggest enhanced immune capacities of mice as a consequence of DM treatment which may be related to whey proteins in DM. Fibrinogen β chain, annexin A1, and Toll-like receptors, which all are components of whey proteins, play roles in the first line of defense during natural immunity (19). Furthermore, DM is rich in lactoferrin (20) which demonstrates diverse biological activities (21) and is readily digestible by gastric fluid and duodenal digestive enzymes (22). These factors may contribute to the regulatory effects of DM on the immune system. DM also is rich in immunoglobulins (23) which also may impact immunoregulation. The thymus is the largest peripheral immune organ in animals and plays a key role in adaptive immunity. Here, mice showed an increased thymus index after intragastric administration of DM which is another indicator that DM enhances immune functions.

Diverse metabolites regulate the functions of immune cells thereby modifying immune capacity (24). L-valine promotes the activation of PI3K/Akt1 in macrophages (24), which serves as an upstream activating factor of mTORC1 (25). This activation subsequently stimulates the mTORC1/S6K signaling pathway to enhance IL-12 production (26), thereby regulating immune responses. In our study, we observed a significant decrease in serum L-valine levels in mice from the DM group which suggests that the mTORC1/S6K signaling pathway may be inhibited in these mice, leading to a reduction in IL-12 production with a consequentl enhancement of immune function. However, we did not measure the concentration of IL-12. Nitric oxide serves as a signaling messenger that improves immune cell activity (27, 28). L-valine stimulates the production of nitric oxide by inhibiting arginase activities (24, 29). The reduction in L-valine levels observed in our study indicates that feeding with DM may suppress nitric oxide production, thereby mitigating inflammation. In addition, nicotinamide is an important factor in immune regulation that dampens the production of pro-inflammatory cytokines by suppressing the expression of cyclooxygenase-2 (COX-2) in macrophages and inhibiting the production of prostaglandin E2 (PGE2) (30). Nicotinamide levels were significantly reduced in mice that were administered DM which demonstrated that DM may contribute to the reduction of pro-inflammatory cytokine production. Nicotinamide is a precursor of nicotinamide adenine dinucleotide (NAD+) (31) which is a crucial cofactor in diverse metabolic networks, including glycolysis, the tricarboxylic acid cycle, and oxidative phosphorylation (32). The energy required by immune-related macrophages primarily comes from glycolysis and oxidative phosphorylation (33). Thus, nicotinamide, as the principal source of NAD^+^ (34), exerts an important role in immune function regulation. Estrogen receptors are activated by binding of estradiol which modulates gene transcription and expression. Thus, estradiol activates downstream signaling pathways and regulates gene expression thereby modifying immune functions (35). The level of β-estradiol was reduced in mice that were fed DM which may be attributable to the entry of β-estradiol into the body which activated signaling pathways and thus improves the immune function of the animals. Moreover, our findings indicated that nicotinamide levels were significantly decreased in the DM group which suggests that DM may contribute reduced production of pro-inflammatory cytokines (36). In addition, DM regulates gene expression (37) which may be attributable to metabolites such as β-estradiol. Further pathway analysis of mice treated with DM demonstrated that L-valine is enriched via the valine, leucine, and isoleucine biosynthesis and degradation pathways. Moreover, TNF-α and IL-6 activate branched-chain α-ketoacid dehydrogenase to enhance valine metabolism (38) which is boosted significantly in the liver during inflammatory responses. Mice that were administered DM exhibited significantly elevated levels of TNF-α and IL-6 which indicates increased decomposition of L-valine and leads to a decrease in plasma L-valine concentrations. This decrease also may elucidate why plasma L-valine levels decrease following camel milk feeding whereas immunity improves.

*Lactobacillus* regulates the immune system response. The bacterium promotes local mucosal immune reactions and stimulates the production of B cells secreted by IgA on the intestinal mucosa (39). In addition, *Lactobacillus* increases the concentrations of certain cytokines, including IL-4 (40), thereby exerting a pronounced immunoenhancement effect. Mixing of *Lactobacillus* in swine feed raised the level of INF-γ in swine serum (41) which emphasizes the effect of this bacterium in regulating immunity. Here, mice that were administered DM had an increased abundance of *Lactobacillus* in feces, as well as an elevated plasma concentration of IL-6 which may reflect the immunoregulatory properties of *Lactobacillus* in the murine gut after intragastric administration of DM. Furthermore, *L. intestinalis* and *L. murinus* enhance the production of TNF-α in mice (42). Here, mice in the DM group exhibited increased abundance of these two species, as well as elevated TNF-α levels, compared to animals in the DW group. Thus, in addition to the effects of small molecules described above, DM may regulate the immunity of mice via modulation of the gut microbiome.

Coenzyme Q has the potential to activate the immune system by affecting pro-inflammatory markers, as well as the function of mitochondria, lysosomes, and peroxisomes (43). These effects may be attributed to the antioxidant properties of the enzyme which combats free radicals and prevents lipid peroxidation (44). In addition, an increased concentration of coenzyme Q10 mitigates the effects of coenzyme Q2 deficiency on the immune system (45). Correlation analysis of metabolomics and microbiomics data here showed that *Falsiroseomonas* and *Salipiger* are both correlated with coenzyme Q in mice administered DM. Furthermore, coenzyme Q is advantageous for improving immune function (46, 47) and alleviating oxidative stress (46, 48). Thus, DM may affect coenzyme Q levels in mouse plasma by regulating the levels of *Falsiroseomonas* and *Salipiger* and accompanying metabolites, thereby further impacting the mouse immune system.

## 5 Conclusions

This study analyzed the influence of intragastric administration of DM on the gut microbiome, plasma metabolites, and immune cytokine levels of mice. The animals exhibited increased levels of immune cytokines in liver, showed modulated gut microbiome compositions, and displayed altered metabolites in plasma. Thus, DM regulates immunity by transforming gut microbiomes and plasma metabolites. These findings will facilitate the development of methods for improving health by leveraging the intervention and regulation of DM on gut microbiomes.

## Data Availability

The gut microbiomics datasets presented in this study can be found in online repositories. The names of the repository/repositories and accession number(s) can be found at: Sequence Read Archive (PRJNA1218236).
