# Supplementary material for: Impact of intragastric administration of donkey milk on mouse immunity utilizing gut microbiomics and plasma metabolomics
Source: Front Vet Sci. 2025 Mar 12;12:1486406. doi: 10.3389/fvets.2025.1486406 (PMC11938429; doi:10.3389/fvets.2025.1486406)
Supplement: Supplementary file 1 [file Table_1.doc]

Table S1 Nutritional composition of donkey milk

| Nutritional composition | Protein | Fat | Lactose | Mineral |
| --- | --- | --- | --- | --- |
| Content(%) | 1.45 | 1.76 | 7.08 | 0.36 |

Table S2 Nutrients of Feed

| Index | Moisture | Crude protein | Crude fat | Crude Fiber | Ash | Ca | P |
| --- | --- | --- | --- | --- | --- | --- | --- |
| Content(%) | 9.7 | 19.48 | 4.6 | 2.0 | 5.6 | 1.34 | 0.82 |

Table S3 Mouse weight information

| Day | 1^st^ day | 8^th^ day | 15^th^ day | 22^nd^ day | 29^th^ day |
| --- | --- | --- | --- | --- | --- |
| DW (g) | 23.57±0.25 | 26.69±0.57 | 29.34±0.85 | 30.50±0.85 | 31.97±0.80 |
| DM (g) | 23.49±0.20 | 26.25±0.35 | 28.77±0.86 | 30.44±0.72 | 31.92±0.69 |

Data is presented in mean ± SD format.
